# Length of gestation and birth weight are associated with indices of combined kidney biomarkers in early childhood

**DOI:** 10.1371/journal.pone.0227219

**Published:** 2019-12-31

**Authors:** Yuri Levin-Schwartz, Paul Curtin, Katherine Svensson, Nicolas F. Fernandez, Seunghee Kim-Schulze, Gleicy M. Hair, Daniel Flores, Ivan Pantic, Marcela Tamayo-Ortiz, María Luisa Pizano-Zárate, Chris Gennings, Lisa M. Satlin, Andrea A. Baccarelli, Martha M. Tellez-Rojo, Robert O. Wright, Alison P. Sanders

**Affiliations:** 1 Department of Environmental Medicine and Public Health, Icahn School of Medicine at Mount Sinai, New York, NY, United States of America; 2 Human Immune Monitoring Center, Icahn School of Medicine at Mount Sinai, New York, NY, United States of America; 3 Department of Oncological Science, Icahn School of Medicine at Mount Sinai, New York, NY, United States of America; 4 Department of Pediatrics, Icahn School of Medicine at Mount Sinai, New York, NY, United States of America; 5 Center for Nutrition and Health Research, National Institute of Public Health, Cuernavaca, Morelos, Mexico; 6 Department of Developmental Neurobiology, National Institute of Perinatology, Mexico City, Mexico; 7 National Council of Science and Technology, Mexico City, Mexico; 8 Division of Community Interventions Research, National Institute of Perinatology, Mexico City, Mexico; 9 Department of Environmental Health Sciences, Columbia University, New York, NY, United States of America; Universidad Nacional Autonoma de Mexico, MEXICO

## Abstract

Infants born prematurely or with low birth weights are more susceptible to kidney dysfunction throughout their lives. Multiple proteins measured in urine are noninvasive biomarkers of subclinical kidney damage, but few studies have examined the joint effects of multiple biomarkers. We conducted an exploratory study of 103 children in the Programing Research in Obesity, Growth, Environment, and Social Stressors (PROGRESS) longitudinal birth cohort, and measured nine proteins selected *a priori* in banked spot urine samples collected at ages 4–6. The goal of our study was to explore the *combined effects* of kidney damage biomarkers previously associated with birth outcomes. To do this, we generated kidney biomarker indices using weighted quantile sum regression and assessed associations with length of gestation or birth weight. A decile increase in each kidney biomarker index was associated with 2-day shorter gestations (β = -2.0, 95% CI: -3.2, -0.9) and 59-gram lower birth weights (β = -58.5, 95% CI: -98.3, -18.7), respectively. Weights highlighting the contributions showed neutrophil gelatinase-associated lipocalin (NGAL) (60%) and osteopontin (19%) contributed most to the index derived for gestational age. NGAL (66%) and beta-2-microglobulin (10%) contributed most to the index derived for birth weight. Joint analyses of multiple kidney biomarkers can provide integrated measures of kidney dysfunction and improved statistical assessments compared to biomarkers assessed individually. Additionally, shorter gestations and lower birth weights may contribute to subclinical kidney damage measurable in childhood.

## Introduction

As nephron development is typically complete around 36 weeks of gestation [1], infants with shorter gestations and lower birth weights are particularly susceptible to disrupted kidney development [2, 3]. In a recent study of over 4 million Swedish birth records, shorter gestations were associated with an increased lifelong risk of chronic kidney disease (CKD) [4]. Moreover, preterm birth and/or low birth weight are associated with a higher risk of acute kidney injury (AKI) [3]. However, the widely utilized index of kidney function, serum creatinine (SCr), is suboptimal for detecting subclinical renal damage as it may not change until 25–50% of kidney function is lost [5]. Thus, in the absence of clinically apparent disease or AKI, new approaches for early detection of kidney damage and risk of CKD are critically needed, particularly among susceptible populations including individuals born early or lower birth weight.

Multiple proteins represent validated or emerging biomarkers of renal damage in children that can be obtained noninvasively, through analysis of spot urine [6, 7]. These include proteins that escape the normally restrictive glomerular filtration barrier (*i*.*e*., albumin and cystatin C) [6, 8], fail to be reabsorbed in the proximal tubule (*i*.*e*., alpha-1-microglobulin (A1M) and beta-2-microglobulin (B2M)) [9, 10], or are released by tubular epithelial cells in response to injury (*i*.*e*., neutrophil gelatinase-associated lipocalin (NGAL), osteopontin (OPN), kidney injury molecule-1 (KIM-1), clusterin, and trefoil factor 3 (TFF-3)) [11–15]. We also note that many of the proteins above, such as albumin, can be non-specific markers of glomerular or tubular injury [7]. Prior studies have proposed that levels of albumin may even be a useful surrogate for nephron number [16, 17].

Multiple kidney proteins can be measured in urine noninvasively, and there is interest in investigating their individual utility as biomarkers to detect renal damage [18], especially among children [19]. Possibly no single protein adequately encapsulates damage to all parts of the kidney [20], and proteins considered jointly may serve as improved assessments for kidney damage [6, 20, 21]. Similarly, nearly all prior studies that assess the impact of early life insults on kidney health in neonates or children using urinary renal biomarkers report levels of individual biomarkers. These studies have reported associations between shorter gestations and higher urinary levels of NGAL [2, 22–24], albumin [16, 22, 24], cystatin C [22, 24], OPN [2, 22, 24], KIM-1 [2, 24] and TFF-3 [24]. Higher levels of NGAL [23], albumin [16, 25], and A1M [26] have also been linked to lower birth weight.

In this work, we set out to improve upon existing studies of individual kidney damage biomarkers by examining the association between *combinations* of nine kidney biomarkers selected *a priori* and two birth outcomes: length of gestation and birth weight. To our knowledge, no prior study has reported the combined effects of multiple kidney damage biomarkers, examined in early childhood, and the association with length of gestation and birth weight. This work is also the first, to our knowledge, to examine kidney biomarker proteins prospectively among a generally healthy population of children. In this work, we selected weighted quantile sum (WQS) regression [27] to account for the correlation among protein levels and created an integrated index comprised of nine kidney biomarkers. In this exploratory study, we demonstrate a promising approach to examine the combined effects of kidney damage biomarkers and their associations with birth outcomes.

## Materials and methods

### Study participants

This study included a subcohort of 103 Mexican children, 4 to 6 years of age, participating in the Programing Research in Obesity, Growth, Environment, and Social Stressors (PROGRESS) longitudinal birth cohort in Mexico City. There were 948 women who delivered a live child into the cohort. For this pilot study, 103 children with residual spot urine samples were randomly selected for retrospective kidney biomarker analyses. The children were healthy and free of kidney or cardiovascular disease assessed by maternal questionnaire. The full details regarding enrollment for the parent cohort have been previously published [28]. Briefly, between 2007 and 2011, women in their second trimester of pregnancy were recruited through the Mexican social security system (Instituto Mexicano del Seguro Social). Women were eligible if they were at least 18 years of age, at fewer than 20 weeks of gestation, free of heart and kidney disease, not using anti-epilepsy drugs or steroids, and not consuming alcohol daily. The PROGRESS study protocols were approved by the institutional review boards (IRBs) of: the Icahn School of Medicine at Mount Sinai (IRB protocol number: 12–00751), Brigham and Women’s Hospital (IRB protocol numbers: 2006-P-001416 and 2006-P-001792), and the Mexican National Institute of Public Health (IRB protocol number: 560). Informed consent was obtained from the mothers prior to collection of the samples and all methods were carried out in accordance with the relevant guidelines and regulations.

### Participant data collection

Demographic and birth outcome information were collected from PROGRESS participants, including child sex, age, and body mass index (BMI) at urine collection as well as maternal report of indoor smoke exposure during pregnancy. Maternal report of indoor smoke exposure was recorded during the second trimester and indicated whether anyone in the household smoked inside the home. BMI was calculated as weight divided by the square of height (kg/m^2^). For regression analyses, BMI was analyzed as a continuous variable due to limited numbers of subjects that were overweight/obese at 4–6 years of age (only 8 participants in this subcohort met the definition of overweight/obese [29]). Gestational age was derived in units of weeks using a combination of two methods, the maternally reported last menstrual period (LMP) to the date of delivery and the Capurro method, an infant physical exam. In general, the LMP estimate was used. However, if the gestational age estimated using the LMP differed by more than three weeks from the gestational age estimated using the Capurro method, the Capurro method-derived estimate was used (n = 4) [30]. Capurro method estimates were interpolated, by using weeks of gestation + 0.5 weeks (imputed mid-week). Birth weight was reported in kg. In our primary analyses, both gestational age and birth weight were analyzed as continuous outcomes. However, for descriptive statistics and secondary analyses, a child was considered preterm if they had a gestational age of less than 37 weeks and a child was considered low birth weight if the birth weight was lower than 2.5 kg. We explored the overlap between the preterm birth and low birth weight groups (S1 Table). In a sensitivity analysis, we excluded infants defined as small-for-gestational age (SGA) based on Fenton percentile. Fenton birth weight percentiles were derived by comparing each child’s birth weight to other infants of similar gestational ages [31], where SGA was defined as Fenton birth weight percentile less than the 10^th^ percentile [31]. The Fenton charts were applied to all infants, not only those who were born preterm. Fenton charts for term children are based upon the WHO Growth Standard [32]. A spot urine sample was obtained when the children were between the ages of 4 to 6 years. Samples were stored at -80°C and shipped to the Mount Sinai Laboratory for Environmental Nephrotoxicology for further analyses. Serum and plasma creatinine were not measured at the age 4–6 year visit; however, for a subset of 68 children followed up to age 8–9, serum and plasma creatinine were measured subsequently at 8–9 years of age. For this subset of subjects with available data at 8–9 years of age, we report the descriptive statistics for creatinine and calculated estimated glomerular filtration rate (eGFR) using the plasma-based Schwartz formula: $36.5\times \frac{h}{P}$, where *h* is the subject’s height in cm and *P* is the plasma creatinine level in μmol/L [33].

### Urinary protein quantitation and normalization

Levels of urinary proteins were determined from spot urine samples. Protein levels were analyzed at the Mount Sinai Human Immune Monitoring Center. The urine soluble kidney biomarkers included: albumin, NGAL, OPN, KIM-1, clusterin, A1M, cystatin C, B2M, and TFF-3. These proteins were selected due to their role as validated or emerging non-invasive biomarkers of renal damage [7, 34] as well as their ability to be measured using the Luminex-multiplex system. Levels of the biomarkers were determined using a bead-based enzyme-linked immunosorbent assay (ELISA) panel by Milliplex xMAP technology (EMD Millipore, Billerica, MA) on Luminex-200 multiplex immunoassay system. Samples were measured in duplicate in three 96 well plates, of which 80 wells were for 40 samples measured in duplicate, 14 wells were for standard curve measurements, and 2 were for assay quality control. Data were analyzed using Milliplex Analyst 5.1 software (EMD Millipore, Billerica, Massachusetts) and Python. Plate-to-plate variation was adjusted via scaling to achieve equal median mean fluorescent intensity (MFI) values across all plates. After normalizing sample MFI measurements in all plates, MFI data were transformed to concentrations using the average of the standard curve measurements in each plate. The inverse sigmoid functions were used to transform these MFI measurements into concentrations. For comparison with other pediatric populations, we report kidney biomarkers as concentrations per volume. However, since WQS data are quantiled, we used the protein MFI values in our statistical analyses. This approach enables the use of all acquired assay data because we retain samples below a level of detection without the need for imputation.

Urinary creatinine concentration was measured at the Mount Sinai Clinical Chemistry Laboratory. Concentrations of creatinine were determined using enzymatic assays as per the manufacturer’s instructions using an Abbott Architect c16000 clinical chemistry analyzer and the ARCHITECT System Software (Abbott Diagnostic, Abbott Park, Illinois). Quality control was performed using the Bio-Rad Liquid Unassayed Multiqual Control and the Bio-Rad Unity Real Time software (Bio-Rad Laboratories, Hercules, California). Conversion to concentrations was performed using the ARCHITECT System software.

### Statistical analyses

#### WQS kidney biomarker index

Detailed explanation of the WQS technique has been published previously [27]. Briefly, WQS is a method to assess the total impact of a mixture of predictors grouped in quantiles to predict their joint impact on an outcome. The method also enables the determination of relative contributions of components within the mixture to the association. WQS inherently integrates complementary information about the components of the mixture, in this case urinary biomarkers [35]. In our application, if we consider N kidney proteins that are grouped into quantiles, *q*_*ij*_, and an outcome of interest, *y*, the general form of the WQS model is

$$g(\mu )\approx \beta_{0}+\beta_{1}\left(\sum_{i=1}^{N}w_{i}q_{ij}\right)+z^{\prime }\phi ,$$

where *g*(*μ*) is the non-linear link function, in this case the identity function, *β*_1_ represents the effect of the mixture, ***z*** is the set of covariates with corresponding regression coefficients represented by ***ϕ***, and *w*_*i*_ are the weights of each protein, estimated using the mean weights across 1,000 datasets composed of bootstrapped samples and constrained to both be non-negative and sum to 1 [27]. Due to these constraints, the weights represent the relative contribution of each protein to the overall association, with a higher weight representing higher relative contribution of the corresponding protein level. All models were adjusted for child sex, age, indoor smoking, and BMI (continuous). We also included creatinine as a covariate to account for the effect of urinary dilution rather than dividing the level of the protein directly by the concentration of creatinine [18]. Despite some debate, currently this is considered the preferred statistical method to account for the effect of urinary dilution rather than dividing the level of the protein by the concentration of creatinine [36–38]. A sensitivity analysis in which protein levels were directly divided by urine creatinine levels resulted in similar findings to those reported in the primary analysis (results not shown).

In this application of WQS, we treat the birth outcome, gestational age or birth weight, as the dependent variable and the set of proteins as the component variables comprising the mixture. This method is a reasonable way to assess the impact of birth outcomes on kidney biomarkers in childhood, since an estimate of an association captures the relationship between two variables, here a birth outcome and the kidney biomarker index, regardless of which variable is specified as the response or predictor [39]. Since we are interested in lower (*i*.*e*., poorer) birth outcomes, we constrain the model to estimate weights such that the association between the index and the birth outcome are nonpositive. Models generated where the direction of the association was constrained to be nonnegative were not significant and are therefore excluded from the results.

#### Preterm birth and low birth weight analyses

Our primary analyses considered gestational age and birth weight as continuous variables. However, in secondary analyses, we performed logistic WQS regression to investigate the renal effects of birth outcomes based upon clinical definitions such as preterm birth (gestational age < 37 weeks; n = 14) and low birth weight (birth weight < 2.5 kg; n = 15). We used logistic WQS regression to determine whether the mixture of kidney biomarkers was related to the odds of preterm status or the odds of low birth weight status. The covariates in the secondary analyses were identical to those in the primary analyses (child sex, age, BMI, maternal report of indoor smoking, and urinary creatinine).

#### Individual proteins adjusted linear regression

In addition to the models estimated using WQS to assess the biomarker mixture, we also performed a linear regression to estimate the association between each individual protein with continuous gestational age or birth weight. In these models, individual protein levels grouped into deciles served as the predictors and covariates were identical to above (child sex, age, BMI, urinary creatinine, and maternal report of indoor smoking). Further, Pearson correlation between each protein and the birth outcomes are reported in S2 Table.

#### Sensitivity analyses

Due to the association between length of gestation and birth weight, we also analyzed our data excluding the 19 SGA infants, *i*.*e*., those with Fenton birth weight percentiles less than the 10^th^ percentile [31]. The SGA infants are more likely to have experienced restricted fetal growth. Excluding SGA infants from analysis enables examination of whether the primary associations may be driven by lower birth weights (*i*.*e*., restricted fetal development in utero) or shorter gestations. We performed linear WQS regression identical to those above.

## Results

### Characteristics of the study population

The demographics of the 103 participants in this study are presented in Table 1. Fifty-five percent of the children were girls and the average age was 4.7 years (range: 4.0 to 6.4 years). Gestational age ranged from 30 weeks to 43 weeks and fourteen children (14%) were born preterm (<37 weeks). Birth weight ranged from 1.05 kg to 3.98 kg; fifteen children (15%) had low birth weights (<2.5 kg). The demographics of this subcohort did not deviate significantly from the parent cohort. The concentrations of urinary creatinine (Table 1) in this population are within normal ranges for children in this age group [36]. The concentrations of NGAL, albumin, KIM-1, cystatin C, and B2M (Table 2) were within normal ranges for this age group [40–43]. For the subset of 68 subjects follow-up to age 8–9, plasma and serum creatinine as well as eGFR levels (Table 1) were all considered normal for this age group [44–47].

**Table 1:** Demographic information for 103 children in the PROGRESS study at 4 to 6 years.

Demographics	Category	N (%)
**Gender**		
	Female	57 (55)
	Male	46 (45)
**Indoor Smoking**		
	Yes	31 (30)
	No	72 (70)
		**Mean ± SD (range)**
**Age at urine collection (years)**		4.7 ± 0.5 (4.0–6.4)
**Gestational age (weeks)**		38.6 ± 1.91 (30.0–43.3)
**Birth weight (kg)**		2.97 ± 0.49 (1.05–3.98)
**Child BMI (kg/m**^**2**^**)**		15.7 ± 1.7 (12.8–21.9)
**Urinary creatinine (mg/dL)**		71.6 ± 34.1 (10.4–156.0)
**Kidney function measures at 8–9 years of age (n = 68)**		
**Serum creatinine (μmol/L)**		38.8 ± 8.3 (23.9–59.2)
**Plasma creatinine (mg/dL)**		0.44 ± 0.09 (0.27–0.67)
**eGFR (mL/min/1.73 m**^**2**^**)**		134.1 ± 29.3 (84.9–206.5)

**Table 2:** Descriptive statistics of each protein measured in children’s urine samples.

Protein	Samples above the limit of detection (%)	Mean ± SD (range) concentration
NGAL (pg/ml)	96 (93%)	29.6 ± 98.2 (0.36, 614)
Albumin (ng/ml)	86 (83%)	17.1 ± 22.3 (1.34, 151)
Clusterin (ng/ml)	89 (86%)	0.37 ± 0.45 (0.04, 2.15)
Cystatin C (pg/ml)	67 (65%)	7.45 ± 8.14 (3.06, 39.9)
OPN (ng/ml)	96 (93%)	0.38 ± 0.44 (0.01, 2.57)
A1M (pg/ml)	61 (59%)	216 ± 129 (96.6, 760)
B2M (μg/ml)	66 (64%)	0.23 ± 0.36 (0.01, 2.66)
KIM-1 (ng/ml)	80 (78%)	0.41 ± 0.57 (0.03, 2.77)
TFF-3 (ng/ml)	103 (100%)	13.8 ± 6.87 (3.78, 47.7)

### Kidney biomarker index was associated with length of gestation

**Fig 1:**
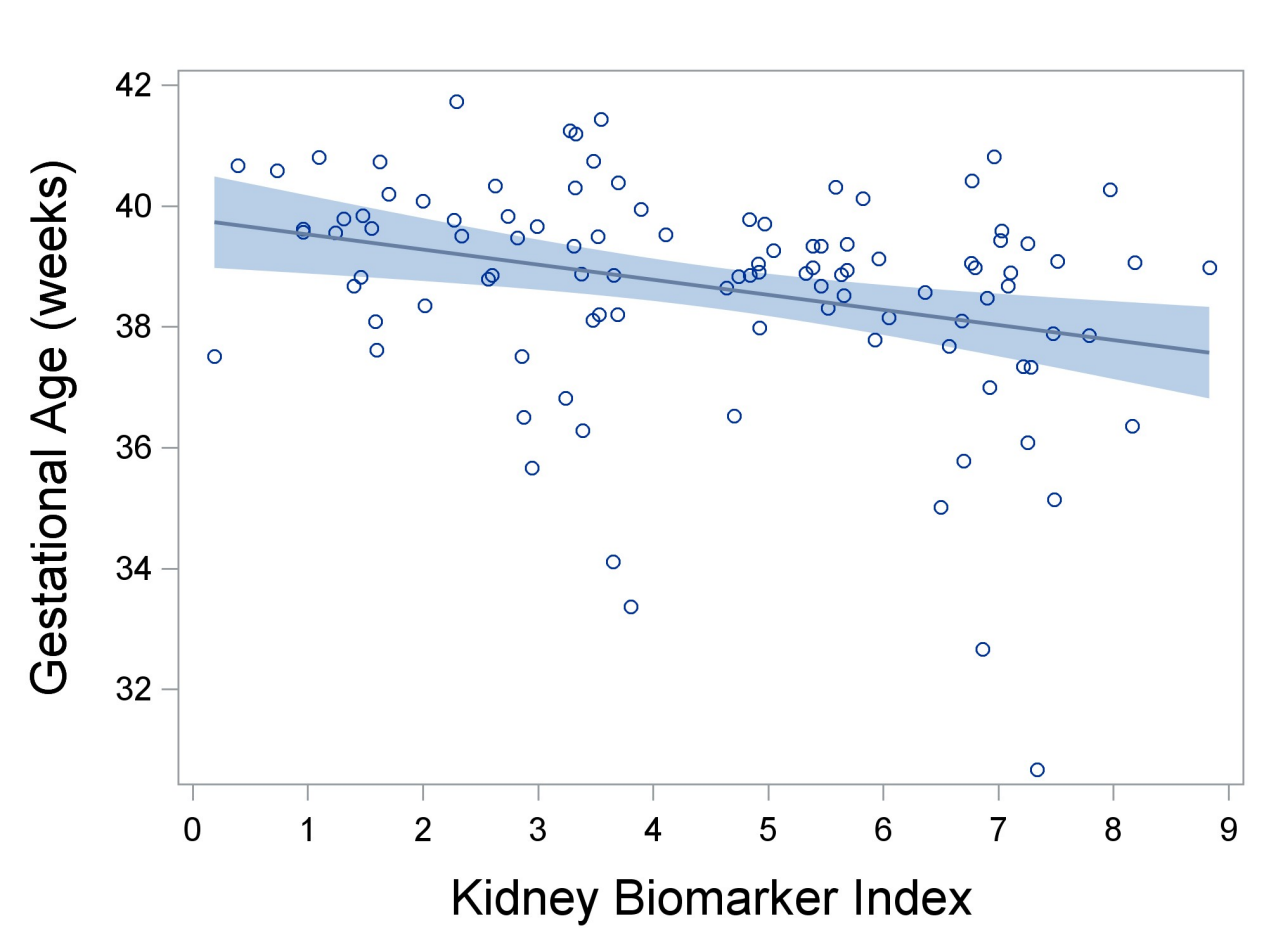
The association between the kidney biomarker index and length of gestation (in weeks) (n = 103). A decile increase in the kidney biomarker index was associated with 0.29-week (2.0-day) shorter gestations (β = -0.29, 95% CI: -0.45, -0.12). The dark blue line is a linear regression with the 95% confidence interval of the mean shown in lighter blue; the circles represent gestational ages. Plotted gestational age values are residuals that account for child sex, age, BMI, indoor smoking, and urinary creatinine.

**Fig 2:**
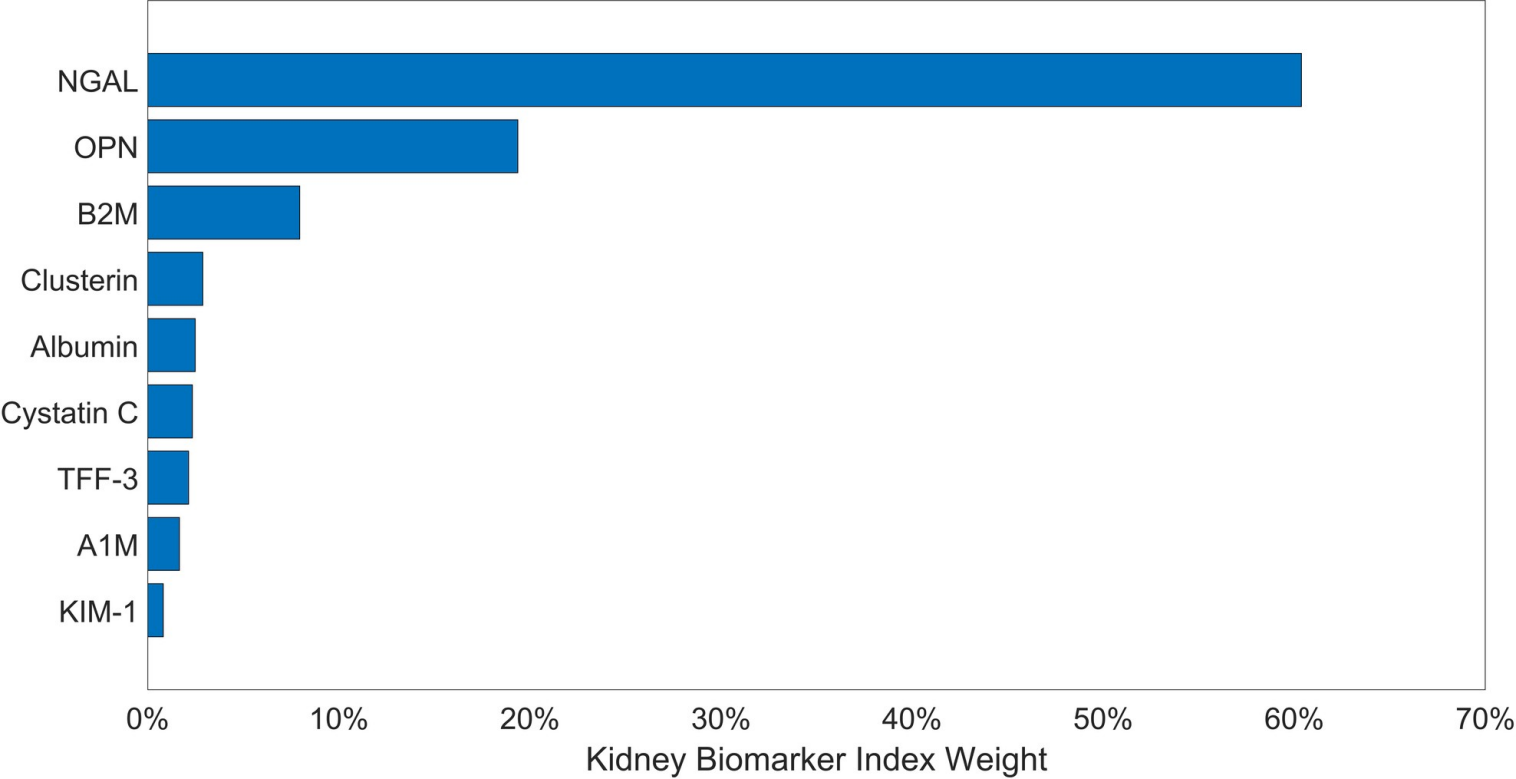
Weights of proteins contributing to the kidney biomarker index estimated for length of gestation. Larger weights indicate greater contribution of the protein to the index.

In adjusted models, the kidney biomarker index was associated with shorter gestations (Fig 1). Specifically, a one decile increase in the index was associated with 2-day shorter gestations (β = -2.0, 95% CI: -3.2, -0.9) shorter gestations (Table 3). The top two protein weights that contributed to this association were NGAL and OPN, which respectively constitute 60% and 19% of the total weight (Fig 2). When NGAL and OPN were assessed individually and not as a mixture, only NGAL was associated with shorter gestations (Table 3). A decile increase in NGAL was associated with 2-day shorter gestations (β = -1.5 95% CI: -2.4, -0.6).

**Table 3:** Gestational age in days per decile increase in individual proteins and the kidney biomarker index. These linear regression models were adjusted for child sex, age, BMI, indoor smoking, and urinary creatinine.

	β (95% CI)	p-value
WQS kidney biomarker index	-2.0 (-3.2, -0.9)	0.0006
**Individual Protein**		
NGAL	-1.5 (-2.4, -0.6)	0.001
Albumin	-0.7 (-1.7, 0.2)	0.14
Clusterin	-0.8 (-1.7, 0.1)	0.10
Cystatin C	-0.4 (-1.3, 0.5)	0.38
OPN	-0.6 (-1.6, 0.3)	0.22
A1M	-0.4 (-1.3, 0.6)	0.45
B2M	-0.8 (-1.7, 0.1)	0.09
KIM-1	-0.7 (-1.6, 0.3)	0.16
TFF-3	0.5 (-0.4, 1.5)	0.28

### Kidney biomarker index was associated with birth weight

**Fig 3:**
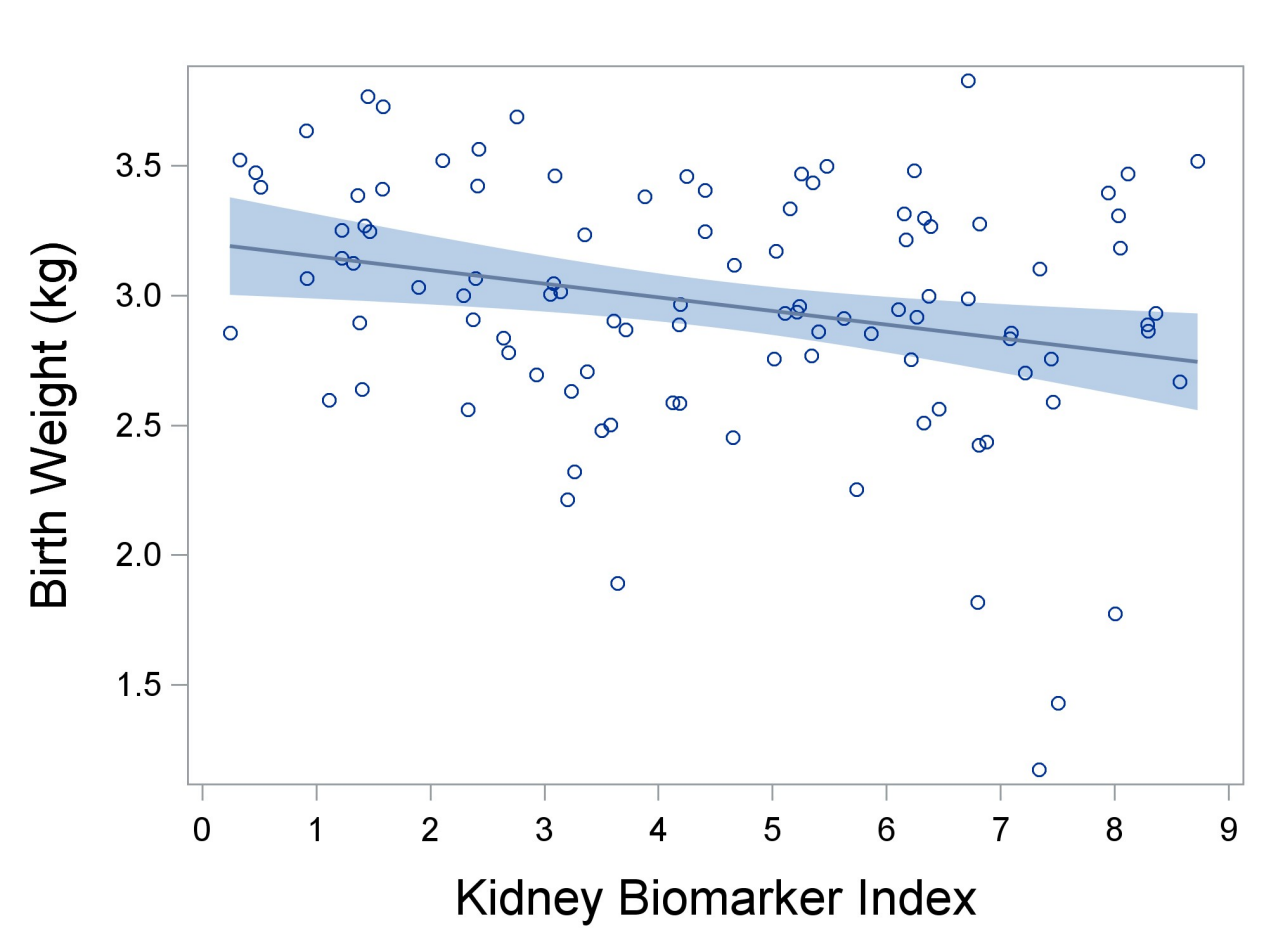
The association between the kidney biomarker index and birth weight (in kilograms) (n = 103). A decile increase in the kidney biomarker index was associated with 0.1-kg (59-gram) lower birth weights (β = -0.06, 95% CI: -0.10, -0.02). The dark blue line is a linear regression with the 95% confidence interval of the mean shown in lighter blue; the circles represent the values of the birth weight. Plotted birth weight values are residuals accounting for child sex, age, BMI, indoor smoking, and urinary creatinine.

**Fig 4:**
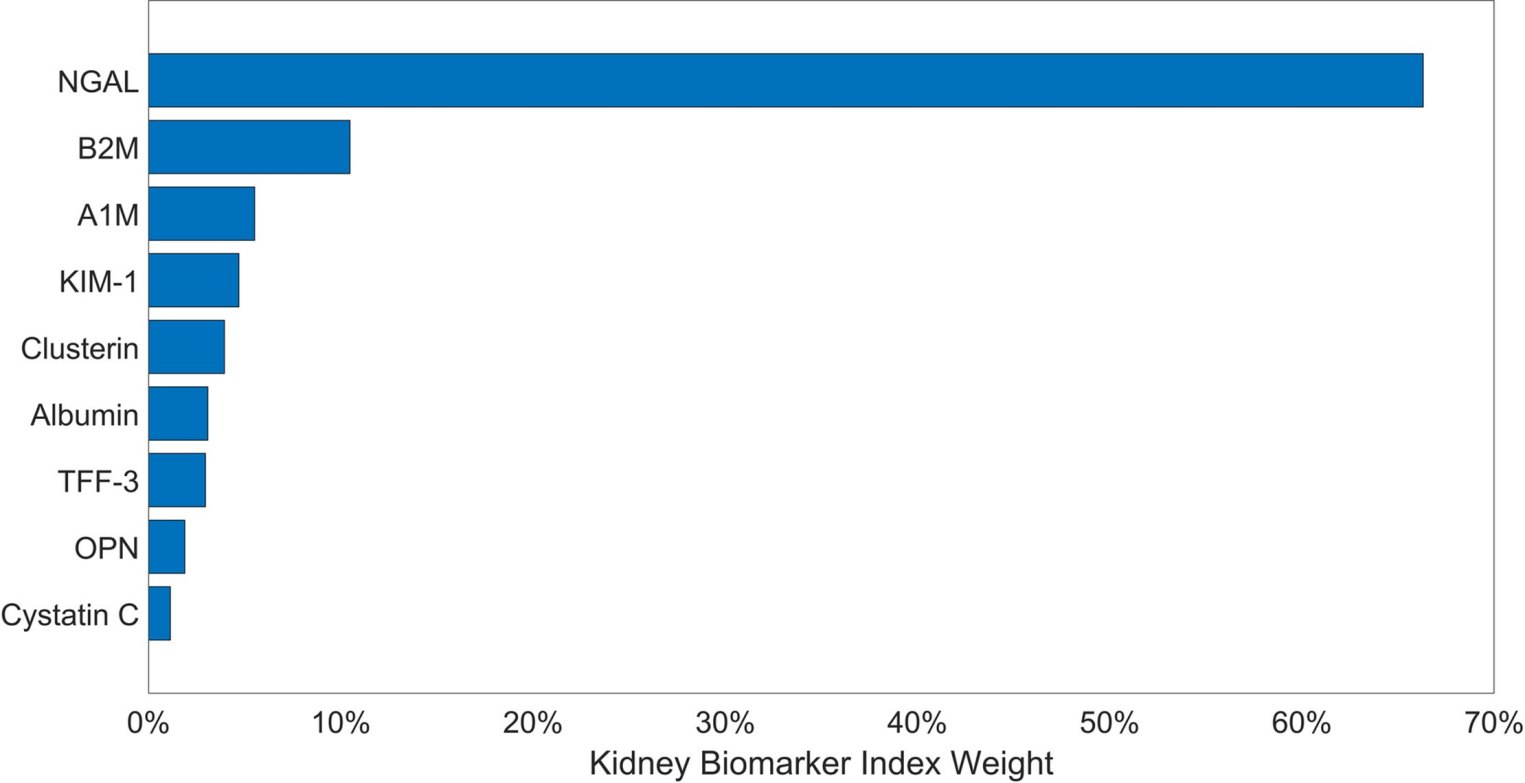
Weights of proteins contributing to the kidney biomarker index estimated for birth weight. Larger weights indicate greater contribution of the protein to the index.

In adjusted models, we found that a one decile increase in the kidney biomarker index was associated with 59-gram lower birth weights (β = 58.5, 95% CI: -98.3, -18.7) (Fig 3 and Table 4). The top two protein weights were NGAL and B2M, which respectively constitute 66% and 10% of the total weight (Fig 4). When assessed individually, we found no association between B2M and birth weight (Table 4), while we observed a significant inverse association between birth weight and NGAL (β: -50.8, 95% CI: -84.3, -17.3). We note that based upon our observed effect sizes, the number samples in our study means that we have 0.92 and 0.76 power to detect effect sizes that were observed for the indices for gestational age and birth weight, respectively.

**Table 4:** Birth weight in grams per decile increase in the individual proteins and kidney biomarker index. These linear regression models were adjusted for child sex, age, BMI, indoor smoking, and urinary creatinine.

	β (95% CI)	p-value
WQS kidney biomarker index	-58.5 (-98.3, -18.7)	0.004
**Individual Protein**		
NGAL	-50.8 (-84.3, -17.3)	0.004
Albumin	-21.4 (-57.6, 14.8)	0.25
Clusterin	-22.9 (-56.4, 10.7)	0.18
Cystatin C	-2.2 (-36.6, 32.3)	0.90
OPN	4.2 (-31.6, 40.0)	0.82
A1M	-14.3 (-49.8, 21.2)	0.43
B2M	-22.0 (-55.4, 11.5)	0.20
KIM-1	-25.6 (-60.0, 8.9)	0.15
TFF-3	18.1 (-17.9, 54.2)	0.33

### Preterm birth and low birth weight secondary analyses

In secondary analyses where the birth outcomes were dichotomized based upon the clinical definitions of preterm birth and low birth weight, kidney biomarker indices estimated for each outcome using logistic regressions showed significant associations. Specifically, an increase of one decile in each corresponding index was associated with a 53.5% increased odds of being born preterm (95% CI: 9.6, 115) and a 67.7% increased odds of being born low birth weight (95% CI: 16.6, 141) (S3 and S4 Tables). For reference, the proportion of subjects that were born preterm is 14% and the number of subjects that were born with low birth weight was 15%. Estimated weights describing the contributions of each protein showed that NGAL (52%), B2M (19%), and OPN (15%) were the largest contributors to the kidney biomarker index associated with preterm birth (S3 Table). This was also the case for the kidney biomarker index associated with gestational age in the main analyses. The top contributors to the index associated with being born low birth weight were NGAL (54%), OPN (20%), and B2M (13%) (S4 Table). Two of these (NGAL and B2M) were also the top contributing proteins to the kidney biomarker index associated with birth weight in the main analyses.

### Sensitivity analyses

In the sensitivity analysis of the relationship between birth weight and the kidney index, *i*.*e*., excluding the 19 SGA infants, the association remained similar. A one decile increase in the index was associated with 50-gram lower birth weights (β = -46.8, 95% CI: -88.7, -4.8) (S5 Table). Also in agreement with the main analysis, the top three contributing proteins were NGAL, A1M, and B2M (S5 Table).

After excluding the 19 SGA infants, we re-analyzed the association between the kidney index biomarker and length of gestation. We found that a one decile increase in the index was associated with 2-day shorter gestations (β = -1.5, 95% CI: -2.7, -0.3) (S6 Table). In agreement with the main analysis, the proteins contributing the largest weights to the index are the same as in this analysis (NGAL, B2M, and OPN), though the values of the estimated weights shifted slightly. In this analysis, NGAL, B2M, and OPN contributed 35%, 27%, and 19% of the weight to the index, respectively (S6 Table).

## Discussion

In a sample of Mexican children free of clinical kidney disease, we report associations between kidney biomarker indices, composed of nine proteins and derived using WQS, and continuous gestational age and birth weight. We identified similar combinations of these proteins associated with increased odds of being born preterm and low birth weight. Our study supports existing literature in identifying higher levels of specific biomarkers of kidney damage (*i*.*e*., NGAL, OPN, and B2M) that are associated with shorter gestations as well as lower birth weights [48–50]. Further, by examining the effects of these proteins in a cumulative mixture model, we provide evidence that their *joint* effects are related to shorter gestations and lower birth weights. Higher levels of all of these proteins have previously been shown to be indicative of kidney damage or dysfunction [18, 51–53]. Therefore, we cautiously interpret our results that shorter gestations and lower birth weights are associated with higher combined protein levels as indicating possible *subclinical* renal damage that is measurable in childhood as early as 4 to 6 years of age. Longitudinal studies are needed to determine whether these changes lead to later life clinically apparent renal disease or dysfunction.

In this study, NGAL and OPN contributed the highest weights in the index associated with shorter gestations, while NGAL and B2M contributed the highest weights in the index associated with lower birth weights. Additionally, these proteins contributed most to the corresponding indices associated with being born preterm and low birth weight. Higher levels of NGAL have been associated with shorter gestations and lower birth weights previously when studied individually [2, 22–24]. Associations between higher levels of OPN in urine and shorter gestations have also been previously reported [2, 22, 24], however to our knowledge, no studies have highlighted an association between B2M and lower birth weights. NGAL is linked to innate immunity to bacterial infection and is induced in regenerating and recovering kidney tubule cells [54]. OPN is associated with tubular repair [12] and B2M is associated with proximal tubular damage [49]. Tubular damage has been noted in both infants that have been born preterm [55] and with low birth weight [56].

Though other studies have found associations between the levels of the kidney biomarker proteins and gestational age as well as birth weight [2, 16, 22–26], we found that only NGAL, when analyzed individually and not in a mixture, was significantly associated with shorter gestations and lower birth weights. Our lack of significant associations with proteins other than NGAL may differ from prior studies due to a number of reasons including study location and demographics, protein quantitation method, and age at urine sample collection among others. It is notable that nearly all prior studies included neonates. The differences in protein levels between children born preterm or low birth weight and those born term and normal birth weight are more apparent after birth and decrease over time [22], potentially due to the kidney repair and maturation that continues after birth. An advantage of using mixtures approaches like WQS is that we can simultaneously evaluate the combined effect of multiple chemicals, while retaining the capacity to assess the discrete effects of each component contributing to the mixture.

In studies of premature neonates, levels of urine biomarkers are often higher due to immature tubular transport mechanisms that impair protein reabsorption [24]. Here, we report altered urine protein levels, and their combinations, persist until 4 to 6 years of age. Since NGAL, OPN, and B2M are indicators of tubular damage and also contributed the highest weights to the two indices, our findings support the previous findings of disrupted tubular function in children with shorter gestations and born with lower birth weights may persist until 4 to 6 years of age [55, 56]. Our results suggest a possible pathway, i.e., impacted tubular transport mechanisms, that may help to explain why preterm birth has been linked to an increased risk of CKD [4]. We posit that this disruption may result from changes that occur *in utero* during critical periods of renal maturation and development [55, 57]. However, studies to replicate these findings and examine underlying mechanisms are needed.

Our study had multiple strengths. The PROGRESS participants are healthy children free of cardiovascular or kidney diseases. Our findings demonstrate associations between birth outcomes and levels of kidney biomarkers at 4 to 6 years of age, a later life stage than the majority of studies, which have assessed neonatal kidney damage. We modeled the associations between the proteins and the birth outcomes using two modeling techniques, linear and logistic WQS regressions, and reached similar conclusions in terms of the proteins that drive the associations, thus increasing our confidence in primary analyses and results. However, some biologically relevant information may be lost in logistic regressions analyses due to the dichotomization of the outcomes. The proteins selected in this study are validated or emerging biomarkers of renal damage or dysfunction, reflecting the glomerular filtration barrier, absorption of the proximal tubule, or non-specific responses to renal damage. Because we used biomarkers that reflect different aspects of kidney damage we did not make *a priori* hypotheses regarding which individual biomarkers or forms of damage would be related to length of gestation or birth weight. Instead, we studied the relationship between birth outcomes and an integrated measure of kidney damage, encompassing multiple aspects of renal damage or dysfunction, while allowing the model to empirically determine each biomarker’s relative contribution to the two indices. Notably, our WQS modeling approach directly addresses the impact of a birth outcome on biomarker levels, and further accounts for age-associated differences through covariate adjustment, an important consideration for assessments of biomarker performance in children and neonates [58]. WQS inherently utilizes the complementary information provided by each protein while alleviating issues of collinearity. A strength of this method is that the proteins are evaluated in the mixed context in which they occur, allowing the opportunity to evaluate the effects of individual proteins in the context of potential additive or combinatorial effects. Additionally, the weights estimated by WQS provide information about the proteins as having relatively higher or lower contributions to the association between the index and the birth outcomes. Information about the weights of the proteins may provide information about potential mechanisms of damage that may be considered in future mechanistic studies of how the *in utero* growth environment affects kidney development. Therefore, future studies and diagnostic strategies in subjects of all ages may wish to consider an analytical strategy that can similarly evaluate additive effects of urinary proteins or other biomarkers, as were observed in these analyses. Indeed, as the index components and corresponding weights may change depending on population characteristics as well as the exposure or outcome of interest, statistical approaches similar to WQS rather than these specific combinations of biomarkers may prove to be the most useful in future studies.

Our study had limitations. We measured and constructed our indices using nine biomarkers selected *a priori*. Reference values for many of these biomarkers for this age group are unavailable, making comparisons to other populations difficult. Since we did not collect empirical clinical information about the renal function of the participants, we are unable to provide clinical conclusions about the relationship between the kidney biomarker indices and renal function. Our analyses utilized pre-collected spot urines due to the retrospective nature of this exploratory study and we did not have information on 24-hour creatinine clearance. Gold standard measures of nephron endowment, such as para-aminohippuric acid clearance (PAH clearance) or effective renal plasma flow, were not collected in this healthy population of children; however, they may not be practical for large population-based studies. Although difficult to disentangle, lower birth weight and shorter gestation may share similar pathophysiology in some cases [59, 60]; therefore the two effects estimated in the present study may not be fully independent. The results from our sensitivity analyses suggest that our main analyses may be capturing evidence of an association driven by shorter gestation, rather than lower birth weight. Therefore, our results require validation in clinically-relevant populations. Finally, our sample size was too small to validate the indices using separate participant data, thus the findings may not be generalizable.

## Conclusions

In this study, we demonstrated the utility of applying WQS, an ensemble machine learning method for mixtures analyses, to determine indices, composed of kidney biomarkers measured in children’s urine at 4 to 6 years of age, and assessed their association with gestational age and birth weight. Joint analyses of multiple kidney biomarkers provided an integrated measure of kidney damage and improved statistical assessments compared to biomarkers assessed individually. Our results also suggest that the renal impact of shorter gestation as well as lower birth weight, observed through biomarkers of tubular damage, may be measurable later in childhood. The goal of this work was to expand upon the idea of a single “gold standard” biomarker to detect renal dysfunction and instead demonstrate that biomarker indices formed from multiple urinary proteins may be more effective than individual proteins. As we would anticipate the index components and weights to change depending on population characteristics and exposure/outcome of interest, similar computational approaches rather than the specific combinations of biomarkers highlighted in this study may contribute to improved evaluations of non-invasive renal diagnostic techniques.

## Supporting information

S1 Table Number of children meeting the definition of preterm and/or low birth weight status in this subcohort (n = 103). (XLSX)

S2 Table Pearson correlation, r (*p*-value), between the birth outcomes, gestational age and birth weight, and each kidney biomarker. (XLSX)

S3 Table Comparison of primary (linear WQS regression) and secondary (logistic WQS regression) analyses of kidney biomarker indices estimated for continuous gestational age or dichotomized preterm birth. Models were adjusted for child sex, age, BMI, indoor smoking, and urinary creatinine. Larger weights indicate greater contribution of the protein to the index.
(XLSX)

S4 Table Comparison of primary (linear WQS regression) and secondary (logistic WQS regression) analyses of kidney biomarker indices estimated for continuous birth weight or dichotomized low birth weight. Models were adjusted for child sex, age, BMI, indoor smoking, and urinary creatinine. Larger weights indicate greater contribution of the protein to the index.
(XLSX)

S5 Table Comparison of primary (n = 103) and sensitivity (n = 84, SGA excluded) analyses of kidney biomarker indices estimated for birth weight. Models were adjusted for child sex, age, BMI, indoor smoking, and urinary creatinine. Effect sizes are in the units of kilograms. Larger weights indicate greater contribution of the protein to the index.
(XLSX)

S6 Table Comparison of primary (n = 103) and sensitivity (n = 84, SGA excluded) analyses of kidney biomarker indices estimated for gestational age. Models were adjusted for child sex, age, BMI, indoor smoking, and urinary creatinine. Effect sizes are in the units of weeks. Larger weights indicate greater contribution of the protein to the index.
(XLSX)
